# Double-catheter lavage combined with percutaneous flexible endoscopic debridement for infected pancreatic necrosis failed to percutaneous catheter drainage

**DOI:** 10.1186/s12876-017-0717-3

**Published:** 2017-12-08

**Authors:** Pi Liu, Jun Song, Hua-jing Ke, Nong-hua Lv, Yin Zhu, Hao Zeng, Yong Zhu, Liang Xia, Wen-hua He, Ji Li, Xin Huang, Yu-peng Lei

**Affiliations:** Department of Gastroenterology, The First Affiliated Hospital of Nanchang University, 17 Yongwaizheng Street, Nanchang, Jiangxi 330006 People’s Republic of China

**Keywords:** Infected pancreatic necrosis, Double-catheter lavage, Percutaneous flexible endoscopic debridement, Percutaneous catheter drainage, Open necrosectomy

## Abstract

**Background:**

Infected pancreatic necrosis (IPN) is a serious local complication of acute pancreatitis, with high mortality. Minimally invasive therapy including percutaneous catheter drainage (PCD) has become the preferred method for IPN instead of traditional open necrosectomy. However, the efficacy of double-catheter lavage in combination with percutaneous flexible endoscopic debridement after PCD failure is unknown compared with surgical necrosectomy.

**Methods:**

A total of 27 cases of IPN patients with failure PCD between Jan 2014 and Dec 2015 were enrolled in this retrospective cohort study. Fifteen patients received double-catheter lavage in combination with percutaneous flexible endoscopic debridement, and 12 patients underwent open necrosectomy. The primary endpoint was the composite end point of major complications or death. The secondary endpoint included mortality, major complication rate, ICU admission length of stay, and overall length of stay.

**Results:**

The primary endpoint occurrence rate in double-catheter lavage in combination with percutaneous flexible endoscopic debridement group (8/15, 53%) was significantly lower than that in open necrosectomy group (11/12, 92%) (RR = 1.71, 95% CI = 1.04 – 2.84, *P* < 0.05). Though the mortality between two groups showed no statistical significance (0% vs. 17%, *P* = 0.19), the rate of new-onset multiple organ failure and ICU admission length of stay in the experimental group was significantly lower than that in open necrosectomy group (13% vs. 58%, *P* = 0.04; 0 vs. 17, *P* = 0.02, respectively). Only 40% of patients required ICU admission after percutaneous debridement, which was markedly lower than the patients who underwent surgery (83%; *P* < 0.05).

**Conclusions:**

Double-catheter lavage in combination with percutaneous flexible endoscopic debridement showed superior effectiveness, safety, and convenience in patients with IPN after PCD failure as compared to open necrosectomy.

**Electronic supplementary material:**

The online version of this article (10.1186/s12876-017-0717-3) contains supplementary material, which is available to authorized users.

## Background

Acute pancreatitis (AP) is a serious digestive system disease with a morbidity of 13-45/100,000 [1]. 10–20% patients may develop pancreatic and peripancreatic necrosis, while 40–70% of patients with pancreatic necrosis develop asecondary infection [2]. Infected pancreatic necrosis (IPN)) is a severe local complication of AP with a mortality at about 30% (12–39%) [3–7]. The 2012 Atlanta classification define the local complications of acute pancreatits as acute peripancreatic fluid collection, pancreatic pseudocyst, acute necrotic collection (ANC), and walled-off necrosis (WON), which is classified into aseptic or infectious [8]. WON combined with infection is an important cause of death in the late stage of severe AP.

Open necrosectomy is the traditional treatment method for IPN, which may lead to further tissue damage and bleeding, and promote the inflammatory reaction, resulting in high complication rate (up to 95%) and mortality (~ 40%) [9]. A variety of minimally invasive treatment options for IPN have emerged in the last decade, including percutaneous pigtail tube drainage, percutaneous U tube drainage, double pigtail or metal stents drainage through the stomach, and minimally invasive Step-up approach, etc. Percutaneous pigtail tube drainage became the first-line treatment for IPN because of simple operation, convenience, and small disturbance to the patient. However, it was reported that the success rate of percutaneous drainage therapy for IPN was only 33% [10].

Van Santvoort et al. (2010) demonstrated that the minimally invasive step-up approach showed enhanced efficacy and safety in IPN as compared with open necrosectomy [11]. Although numerous reports suggest good efficacy of endoscopic ultrasonography in IPN treatment, video assisted retroperitoneal debridement or endoscopic transgastric necrosectomy requires significant technical expertise because of its complexity. To help further refine the treatment of this condition, this study evaluated the effectiveness and safety of percutaneous catheter drainage, followed by large aperture double catheter lavage combined with percutaneous flexible endoscopic debridement as compared with open necrosectomy.

## Methods

### Clinical information

**Fig. 1:**
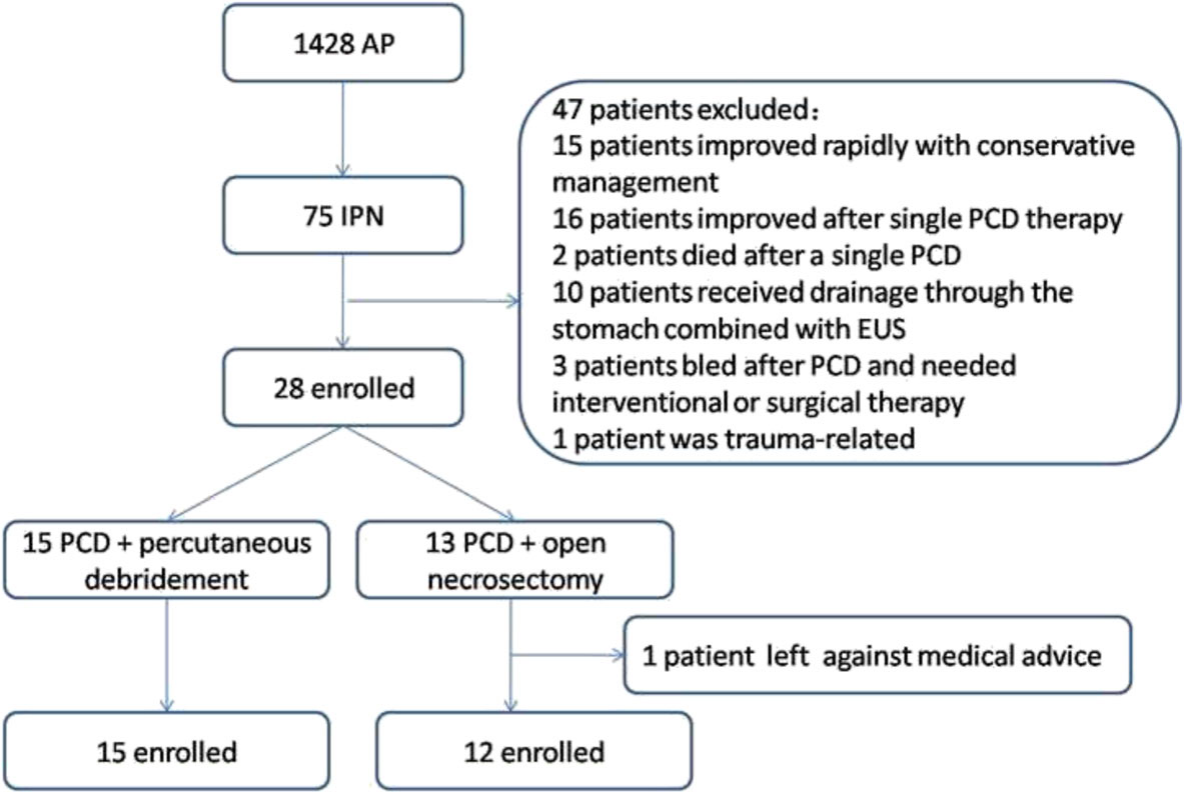
Flow chart

A total of 1428 AP patients were admitted to our hospital, The First Affiliated Hospital of Nanchang University (Nanchang City, PRC), between Jan 1, 2014 and Dec 31, 2015, of which 75 patients were diagnosed as IPN. Twenty-eight patients who failed PCD were enrolled in the retrospective cohort study, one of which was excluded because he left against medical advice. The other 47 patients were excluded as follows: 15 patients improved rapidly with conservative management,16 patients improved after single PCD therapy, two patients died after a single PCD, ten patients received drainage through the stomach combined with endoscopic ultrasonography (EUS), three patients bled after PCD and needed interventional or surgical therapy, and one patient was trauma-related (Fig. 1).

According to the 2012 Atlanta Acute Pancreatitis Classification, IPN diagnosis criteria included: (1) Contrast-enhanced CT showed extraluminal gas in the pancreatic and/or peripancreatic tissue. (2) Percutaneous, image-guided, fine-needle aspiration (FNA) was positive for bacteria and/or fungi on Gram stain and culture [3]. Patients who were considered as PCD failures met the following criteria: (1) Clinical symptoms did not improve after more than 1 week of double catheter lavage and drainage as evidenced by fever, sepsis, and outflow obstruction, etc.; (2) a less than 75% reduction in collecs showed no statistical significantion size after 10 to 14 days’ lavage and drainage [12]. Patients with the diagnosis of infected pancreatic necroses were enrolled after PCD failure.

Exclusion criteria included: (1) Patients with severe heart, lung, liver, or CNS disease, coagulopathies and other medical conditions that would preclude endoscopic or surgical treatment; (2). Pregnant or lactating women; (3) Drainage through the stomach; (4) Bleeding after PCD that required interventional or surgical treatment; (5) Refusal to sign the informed consent; (6) Discharge against medical advice; and (7) Patients who were trauma-related.

The trial was performed in a large pancreatitis center in the First Affiliated Hospital of Nanchang University, Jiangxi Province, China. The study was performed in accordance with the principles of the Declaration of Helsinki. The Medical Ethics Committee of the First Affiliated Hospital of Nanchang University approved the protocol(Ethical approval No. 2013020).All study participants gave informed consent. Written consent from patients was obtained for this study.

The patient demographics between the two groups showed no statistical significance regarding age, gender, BMI, pancreatic necrosis foci location, and severity, except pathogenesis proportion (Table 1).

**Table 1:** Baseline characteristics

Item	PCD + open necrosectomy (*n* = 12)	PCD+ percutaneous debridement (*n* = 15)	*P* value
Age, median (IQR), year	47 (36–57)	54 (48–59)	0.23
Male (%)	11 (92%)	10 (67%)	0.18
Pathogenesis (%)			0.03
Biliary	4 (33%)	11 (73%)	
Hypertriglyceridemia	5 (42%)	4 (27%)	
Other^a^	3 (25%)	0	
BMI, median (IQR)	24 (22–25)	24 (23–27)	0.50
Severity			
APACHE score, median (IQR)	11 (10–12)	9 (7–12)	0.18
CRP, median (IQR), mg/L	217 (156–294)	182 (143–293)	0.67
CTSI score, median (IQR)	10 (8–10)	10 (8–10)	0.73
Ranson score	2 (2–3)	3 (1–3)	0.72
Necrotic lesions			0.22
Pancreatic head, body, and tail	8 (66%)	7 (47%)	
Pancreatic body and tail	2 (17%)	7 (47%)	
Pancreatic head	2 (17%)	1 (6%)	

*PCD* percutaneous catheter drainage, *BMI* body mass index, *IQR* inter-quartile range, *CRP* C-reactive protein, *APACHE* Acute Physiology and Chronic Health Evaluation, *CTSI* Balthazar CT severity index

^a^Including alcoholic and idiopathic

### Double-catheter lavage in combination with percutaneous flexible endoscopic debridement treatment

**Fig. 2:**
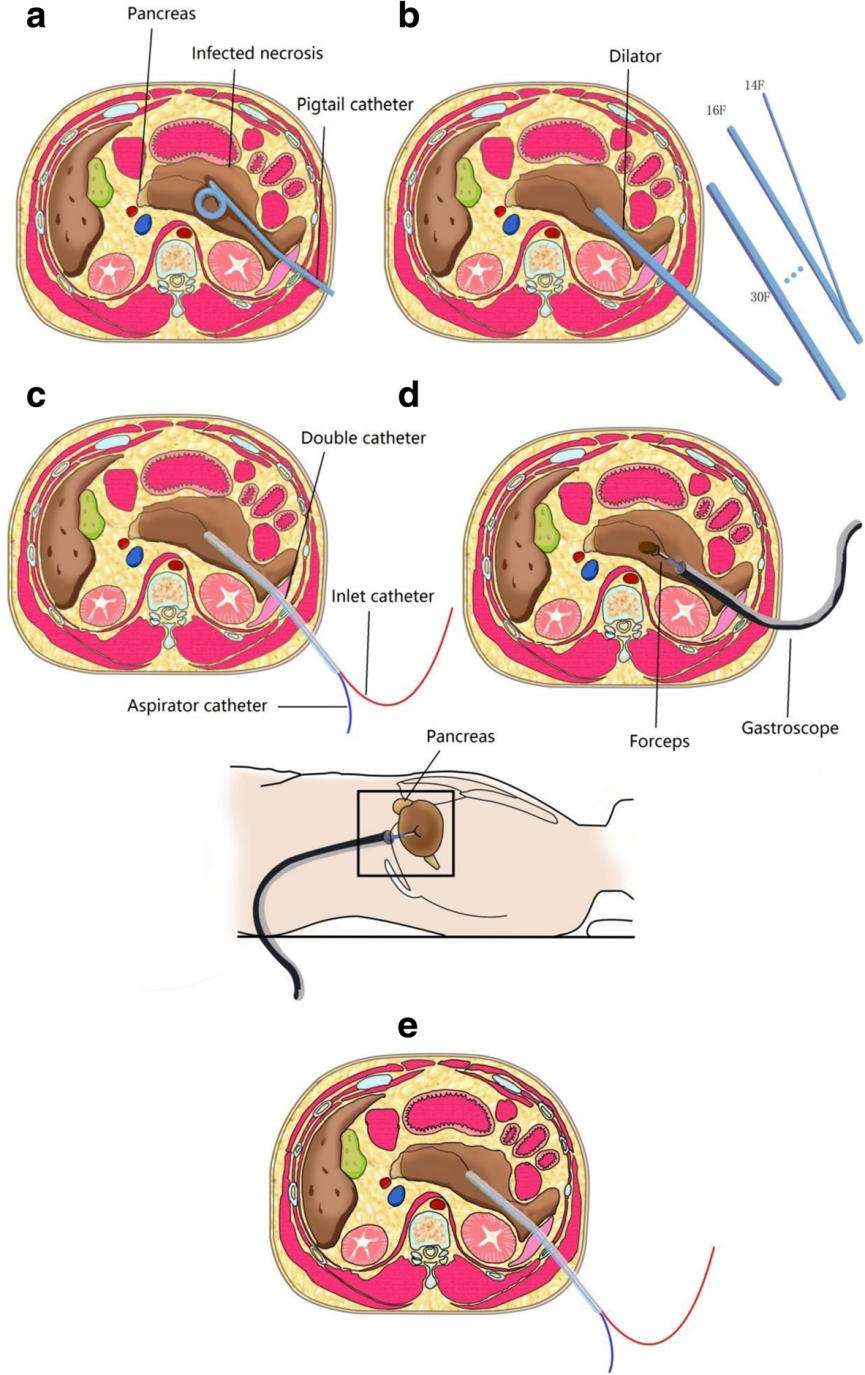
Double-catheter lavage in combination with gastroscopic debridement treatment: **a** A percutaneous 8 to14 French pigtail catheter is placed in the peripancreatic collection under guidance of CT or ultrasound; **b**-**c** A double catheter is placed for continuous irrigation and drainage after the sinus tract was expanded using 14–30Fr dilators; **d** Gastroscopic debridement: the necrosis is removed under direct vision with a long grasping forceps; **e** The double catheter is placed back for continuous irrigation and drainage. The images are designed and drawn by ourselves

**Fig. 3:**
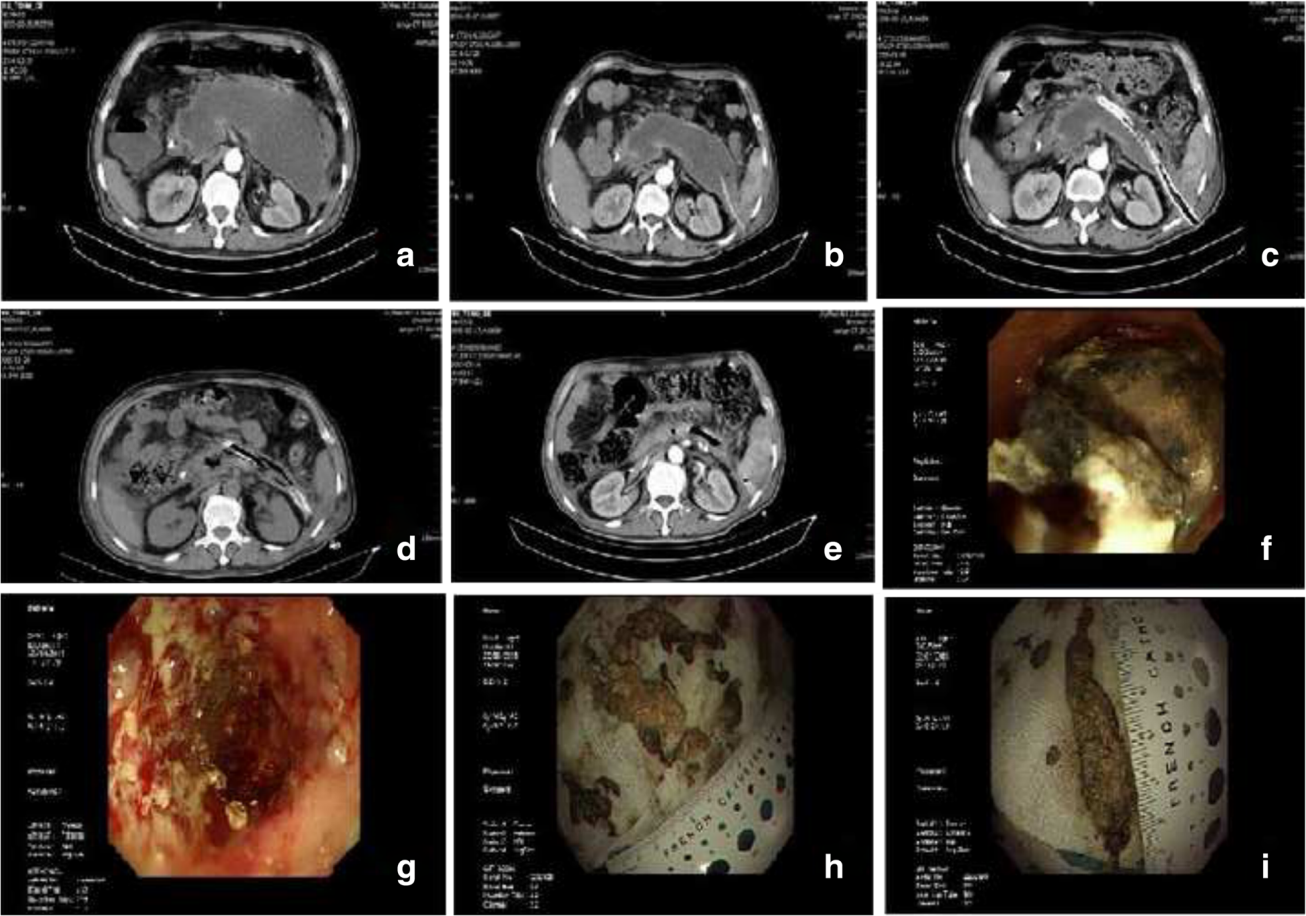
Double-catheter lavage in combination with gastroscopic debridement. **a** before percutaneous debridement. **b** PCD drainage tube placement. **c** double catheter placement. **d** after percutaneous debridement. **e** recovery phase. **f** before debridement. **g** after debridement. **h** and **i**, necrotic tissue

All patients underwent percutaneous puncture by an 18G needle using the seldinger technique under the guidance of ultrasound or CT. A 8 to 14 French pigtail catheter was placed into the peripancreatic collection after the sinus tract was first expanded by a 8–14Fr dilator. A double catheter was placed 1 week later for continuous irrigation and drainage after the sinus tract was expanded using 14–30Fr dilators.(Double catheter is a self-designed large aperture tube, with many holes in its body. One end of the catheter is open; the other end is closed. Two tubes are inserted through the open end, one for irrigation and the other one for drainage. We call them inlet catheter and aspirator catheter respectively. The inlet catheter is longer than aspirator catheter. See Fig. 2c)After 1–2 weeks of lavage, the patients in the experimental group underwent percutaneous flexible endoscopic debridement (Host Olympus 260, gastroscope GIF-Q260J, diameter 9.9 mm, 3.2 mm work channel, UCR carbon dioxide air pump, OFP injection pumps, attachment Olympus hemostatic forceps FD-410QR, and spiral mesh MWB-2X4 and MWB-3X6 from COOK), while the patients in the control group underwent open necrosectomy (Figs. 2 and 3, Video 1–2). The primary endpoint was the composite end point of major complications or death [13]. The secondary endpoint was composed of death rate, major complication rate, postoperative ICU admission time, and hospital stay. The main complications included new-onset organ failure, multiple-organ failure, intestinal fistula, intra-abdominal bleeding, stress ulcer with bleeding, etc. (Table 2).Additional file 1: Video 1.Percutaneous flexible endoscopic debridement, the operation process of double-catheter lavage in combination with percutaneous flexible endoscopic debridement treatment. (see Methods section in more detail). (AVI 317000 kb)
Additional file 2: Video 2.Details on the step-up approach, the operation process of double-catheter lavage in combination with percutaneous flexible endoscopic debridement treatment. (see methods section in more detail). (AVI 521000 kb)

**Table 2:** Definitions of major complication

Major complication	Definition	Comment
New-onset organ failure	New-onset failure (i.e., not present at any time in the 24 h before first intervention); Multiple-organ failure: failure of two or more organs at the same time.	
Organ failure		Adapted from Bradley [24]
Pulmonary failure	PaO2 < 60 mmHg, despite FIO2of 0.30, or need for mechanical ventilation.	
Circulatory failure	Circulatory systolic blood pressure < 90 mmHg, despite adequate fluid resuscitation, or need for inotropic catecholamine support.	
Renal failure	Creatinine level > 177 μmol/l after rehydration or new need for hemofiltration or hemodialysis.	
Systemic complication		Adapted from Bradley
Gastrointestinal bleeding	> 500 ml of blood/24 h	
Intra-abdominal bleeding	Requiring surgical, radiologic, or endoscopic intervention.	
Intestinal fistula	Secretion of fecal material from a percutaneous drain or inflow into the necrotic cavity, either from small or large bowel; confirmed by endscopy, imaging or during surgery.	

### Open necrosectomy

The open necrosectomy, consisted of a laparotomy through bilateral subcostal incisions. After blunt removal of all necrotic tissue, two large-bore drains for postoperative lavage were inserted, and the abdomen was closed.

### Statistical analysis

SPSS 18.0 software was applied for statistical analysis. Measurement data was presented as median and interquartile range (IQR). T test was performed upon normal distribution, while Mann-Whitney U test was performed upon abnormal distribution. Enumeration data was depicted in frequency, and the differences between groups were compared by relative risk (RR), 95% confidence interval, and chi-square test. Fisher’s exact test was used with reference of likelihood ratio chi-square. A *P* < 0.05 was considered as statistical significant.

## Results

As shown in Table 3, The composite primary end point of major complications or death occurred in 11 of 12 patients after open necrosectomy (92%) and in 8 of 15 patients after percutaneous debridement (53%) (RR = 1.71, 95% CI = 1.04–2.84) (*P* = 0.04). For the secondary endpoints, the new organ failure rate was lower in the experimental group (13%) compared to the control group (58%) (*P* < 0.05). Although other major complications including multiple organ failure, intestinal fistula, intra-abdominal bleeding, and stress ulcer with bleeding showed no statistical differences between two groups, their incidences trended lower in the experimental group. This difference was mainly driven by the occurrence of organ failure.

**Table 3:** Primary and secondary end points

Outcome	Open necrosectomy after PCD(*n* = 12)	Percutaneous debridement after PCD (*n* = 15)	Risk ratio (95% CI)	*P* value
Primary composite end point: major complications or death - no. (%)^a^	11 (92%)	8 (53%)	1.71 (1.04–2.84)	0.04
Secondary end points				
Major complication - no. (%)				
New-onset organ failure	7 (58%)	2 (13%)	4.38 (1.11–17.32)	0.04
Multiple-organ failure	3 (25%)	0		0.08
Intestinal fistula	6 (50%)	7 (47%)	1.07 (0.49–2.34)	0.86
Intra-abdominal bleeding	3 (25%)	1 (7%)	3.75 (0.45–31.62)	0.29
Stress ulcer with bleeding	2 (17%)	0		0.19
Other outcome				
Intestinal obstruction	2 (17%)	0		0.19
Cerebral infarction	1 (8%)	0		0.44
Arrhythmia	0	1 (7%)		0.27
Death - no. (%)	2 (17%)	0		0.19
Postoperative ICU admission time				0.02
Median	17	0		
IQR	7–35	0–9		
Days in hospital				0.25
Median	62	52		
IQR	40–73	41–60		

^a^Multiple events in the same patient were considered as one end point

Although no statistical difference was observed in mortality between two groups (*P* = 0.19), two patients died in the control group (17%) because of multiple organ failure and acute respiratory failure, while none died in the experimental group.

The experimental group had a markedly shorter postoperative ICU admission time than the controls (*P* = 0.02). Ten patients were admitted to the ICU in the open necrosectomy group (83%), while only six patients were admitted to the ICU in the experimental group (40%) (P = 0.02). The median hospital stay in the experimental group (52, 41–61 days) was similar to that of the control group (62, 40–73 days).

## Discussion

IPN is one of the leading causes of death in AP. Timely diagnosis and effective treatment for IPN is of great significance to improve the prognosis. In the last 30 years, open necrosectomy has become the gold standard for IPN [2]; however, because the high short-term and long-term complication and fatality rate, minimally invasive techniques, especially the Minimally Invasive Step-up Approach [11], have been introduced in recent years. Acute pancreatitis treatment guidelines published by the American Gastroenterological Association in 2013 recommended that debridement of necrotic tissue be delayed for 4 weeks or later untill the necrotic area is enveloped by a fibrous wall to form WON, which can reduce bleeding and avoid damaging normal pancreatic tissue. Therefore, minimally invasive treatments could be applied first, and the endoscopic or open necrosectomy could be considered unless drainage fails [14]. Zerem E, et al. [15] retrospectively analyzed the outcome of 86 IPN patients managed by Minimally Invasive Step-up Approach, and found that the method was safe and effective, and PCD can allow most patients to avoid surgery. PCD is the minimally invasive method firstly used in the treatment of infectious pancreatitis. Though it exhibited high success rate in treating intra-abdominal abscesses and pancreatic pseudocysts, PCD is still controversial in IPN because of its inability to remove necrotic tissue fragments [16, 17].

A multicenter retrospective study in 2009 revealed that endoscopic transgastric necrosectomy had long-term effectiveness. The initial treatment success rate in 93 IPN patients who underwent endoscopic transgastric necrosectomy was 80%.The complication rate within 30 days was 26%, the mortality rate was 7.5%, and the long-term success rate after an average follow-up of 43 months was 68% [18]. Similarly, another multicenter retrospective study enrolled 104 IPN patients who underwent endoscopic transgastric necrosectomy and showed a success rate of 91%, a complication rate at 14%, and 5% mortality [19]. Compared with open necrosectomy, endoscopic transgastric necrosectomy was more efficacious with lower morbidity and mortality. A randomized, controlled study in 2012 likewise demonstrated that endoscopic transgastric necrosectomy was associated with lower mortality (10% vs 40%), a lower new multiple organ failure rate (0% vs 50%), and lower pancreatic fistula rate (10% vs 70%) as compared to open necrosectomy [20].

Despite the success of endoscopic transgastricnecrosectomy, in the cases after the PCD treatment failure, percutaneous endoscopic debridement was more convenient than endoscopic transgastric necrosectomy. Firstly, endoscopic transgastric necrosectomy requires complex gastric or duodenal puncture and fenestration, while percutaneous endoscopic debridement can use the established drainage channel for exploration and further necrotic tissue removal with no need of advanced endoscopist. Secondly, the former requires general anesthesia, leading to longer procedure times and higher costs, whereas the latter can be accomplished with moderate sedation. Finally, the condition of endoscopic transgastric necrosectomy is limited to necrotic areas adjacent to the stomach or duodenum, while percutaneous endoscopic debridement can reach farther necrotic lesions in the abdominal cavity.

Though percutaneous endoscopic debridement is safe and effective, it still lacks supporting evidence at present. A large single center study in 2016 reported its success rate in 12 patients as high as 92%, a complication rate of 8%, and no procedure-related deaths [21]. Another multicenter study in India also confirmed the safety and efficiency of percutaneous endoscopic debridement. Among 15 IPN patients who underwent endoscopic percutaneous debridement, 14 cases improved, two cases exhibited complications (self-limited bleeding and pancreatic fistula), and there was one fatality [22].

This current study found that: (1) No air embolism occurred in the experimental group. It may be related to stable antrum and gap between sinus tract and endoscopy in percutaneous debridement process, which made the necrotic cavity connected to the outside, declining cavity pressure. In addition, Carbon dioxide is pumped in to expand the space for the instrument and prevent the occurrence of air embolism. (2) Fewer concurrent bleeding, which may be associated with waterflood pump irrigation and avoid of strong clearance of the lesion tissue adhere to the wall. (3) No pancreatic fistula occurred, as most patients with intestinal fistula can be self-healed by inserting thinner rubber drainage. (4) Fewer times of percutaneous debridement (1–2), but longer time duration of each drainage (about 2 h). (5) Our flexible endoscope can be inserted into the anfractuous sinus tract to remove more necrosis under direct vision.

To sum up, PCD is characterized as simple, convenient, larger approach choice, early application to reduce the necrotic cavity pressure and prevent bacterial toxins absorbed into the blood, and improving the systemic inflammatory response syndrome and sepsis [23]. Gastroscope could be applied through the established drainage channel for exploration and removal of necrotic tissue once PCD failure. Follow the principle of minimally invasive step-up approach, this study adopted double catheter lavage in combination with percutaneous flexible endoscopic debridement for IPN treatment. It can reduce the primary endpoint indicators (the composite end point of major complications or death), new organ failure rate, and postoperative ICU admission time compared with open necrosectomy, revealing its safety and effectiveness. Moreover, endoscopic transgastric necrosectomy was preferred when the necrotic lesions was adjacent to stomach or duodenum, which can reduce intestinal canal and vascular damage. Surgical treatment should be chose according to the clinical characteristics and lesions anatomical position. There are many drawbacks in this study: (1) It was a retrospective cohort study in small scale. (2) The constituent ratio of causes was different and there was one patient automatic discharge. (3) The lack of follow-up data. Percutaneous debridement is an effective method for IPN, future large scaled randomized controlled study should be carried out to provide the evidence of evidence-based medicine.

## Conclusions

Our data suggest that double-catheter lavage in combination with percutaneous flexible endoscopic debridement may show superior effectiveness, safety, and convenience in patients with IPN after PCD failure as compared to open necrosectomy. Although the series is small, there is merit in replication of the finding that minimal access approaches confer advantages over the open approach. Our study may provide a new idea for treatment of IPN.

## Abbreviations

ANC: Acute necrotic collection
AP: Acute pancreatitis
EUS: Endoscopic ultrasonography
IPN: Infected pancreatic necrosis
IQR: Interquartile range
PCD: Percutaneous catheter drainage
RR: Relative risk
WON: Walled-off necrosis
